# Changes in Visual Evoked Potential and Optical Coherence Tomography in Parkinson's Disease: A Systematic Review and Meta-Analysis

**DOI:** 10.1155/padi/2386302

**Published:** 2025-09-23

**Authors:** Zahra Hemmatian, Javad Heravian Shandiz, Ali Shoeibi, Nasser Shoeibi, Reyhane Shariati, Batool Haghighi, Firozeh Fereydouni, Negareh Yazdani

**Affiliations:** ^1^Student Research Committee, Mashhad University of Medical Sciences, Mashhad, Iran; ^2^Department of Optometry, School of Paramedical Sciences, Mashhad University of Medical Sciences, Mashhad, Iran; ^3^Refractive Errors Research Center, Mashhad University of Medical Sciences, Mashhad, Iran; ^4^Department of Neurology, School of Medicine, Mashhad University of Medical Sciences, Mashhad, Iran; ^5^Eye Research Centre, Mashhad University of Medical Sciences, Mashhad, Iran; ^6^Rehabilitation Sciences Research Center, Zahedan University Medical Sciences, Zahedan, Iran

**Keywords:** optical coherence tomography, Parkinson's disease, visual evoked potential

## Abstract

**Background:** Previous studies revealed that optical coherence tomography (OCT) and visual evoked potential (VEP) were impaired in patients with Parkinson's disease (PD), but the results were inconsistent; in this meta-analysis, we tried to answer this issue by including studies that performed these two tests on the same sample size.

**Methods:** PubMed, Scopus, Cochrane, and Google Scholar were comprehensively reviewed to retrieve the published studies investigating changes in OCT and VEP responses in PD patients. We analyzed the pooled weighted difference in means between PD patients and healthy controls using the random-effects model.

**Results:** Ten studies were included (12 sets of data), enrolling 337 PD patients and 273 healthy controls. The P100 latency in PD patients was significantly higher compared to healthy controls (difference in means = 6.16, 95% CI: 1.16–11.15, *p*=0.02, *n* = 11). Significant thinning of the retinal nerve fiber layer (difference in means = −4.38, 95% CI: −6.29 to −2.47, *p* ≤ 0.001, *n* = 11) was observed in the PD eyes compared to the healthy subjects. However, no statistically significant difference was found in the means of P100 amplitude (*p*=0.06) and the average central foveal thickness (*p*=0.08) between PD patients and the control group. There was a significant negative correlation between RNFL weighted mean difference and P100 latency (*r* = −0.65, *p* ≤ 0.001) in all subjects.

**Conclusions:** Our results confirmed that Parkinson's patients showed significant thinning of RNFL thickness and prolonged P100 latency time.

## 1. Introduction

Parkinson's disease (PD) is a progressive neurodegenerative disorder characterized by tremor, rigidity, bradykinesia, slowed movement, and an imbalance of the autonomic nervous system [1, 2]. PD has been known as the most common cause of movement disability in the elderly population [3], especially those aged 65 and above [4]. The pathophysiology of PD is assumed to be associated with the death of dopaminergic neurons in the substantia nigra [2], leading to decreased dopamine levels in the striatum, which consequently causes functional changes in the basal ganglia [5]. Recent investigations showed that visual processing is affected in PD due to dysfunction in the dopaminergic neural system. Ocular structure and visual function have been proposed as a new potential biomarker for disease evaluation [6], as visual symptoms occur prior to the onset of motor disturbances [7]. It is assumed that alpha-synuclein deposition, dopamine deficiency, and vessel degeneration in the retina could be potential factors leading to visual defects [8–12]. Early detection of PD is challenging because of its late onset and subtle clinical manifestations [1] Therefore, noninvasive imaging techniques that evaluate the retina–cortical dopaminergic pathway, such as visual evoked potential (VEP) mapping [13–17] and optical coherence tomography (OCT), are gaining popularity for assessing the distribution of dopaminergic neurons and visualizing the characteristics of PD tissue [18]. Previous studies have investigated how alterations in retinal neurons, nerve fiber layer, foveal thickness, and retinal ganglion cell layer (GCL) impact the optic nerve in PD patients. However, the results of both VEP and OCT imaging in PD patients are inconsistent, with some studies finding a prolonged latency in VEP response and others showing no significant changes [19–26]. Moreover, some studies reported that retinal nerve fiber layer (RNFL) thickness and GCL measurements showed no significant difference when comparing PD patients with matched healthy subjects [25–28], whereas other studies revealed a thinner RNFL and GCL in PD patients [29–31].

This meta-analysis aimed to consolidate the existing literature pertaining to alterations in VEP and OCT among individuals with PD. The objective was to provide a comprehensive overview of the findings, offering valuable insights for both clinicians and researchers in the field.

## 2. Methods

**Figure 1:**
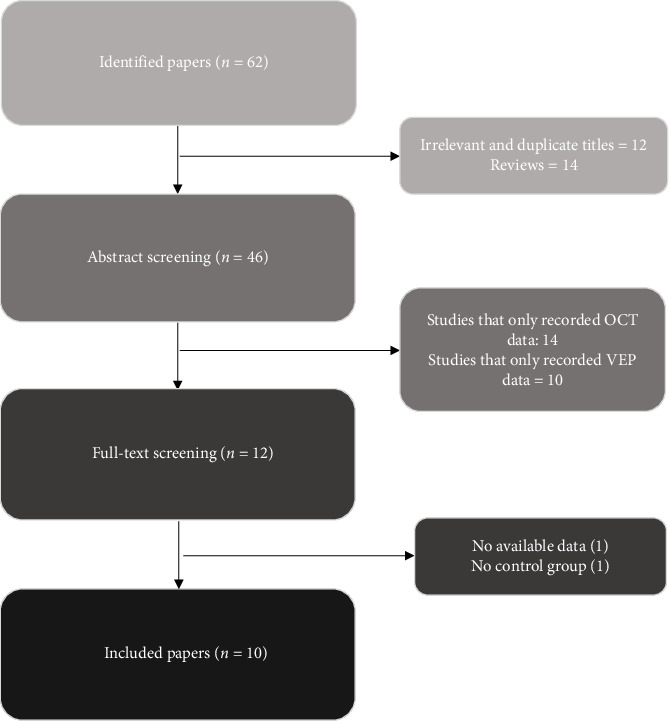
PRISMA flowchart describing the selection of articles.

The present research was carried out in accordance with the PRISMA Guidelines. The following databases were searched up until 30 June 2023: PubMed, Cochrane Library, ScienceDirect, and Google Scholar. The search was not limited to a specific time period or language. We used the following search strategy: “VEP OR Visual Evoked Potential” AND “OCT OR Optical Coherence Tomography,” AND “PD OR Parkinson Disease.” All studies assessing changes in VEP and OCT in PD were included except for the conference abstracts, case reports, and case series and review papers. In addition, reference lists of all publications meeting the inclusion criteria were manually searched to identify any further studies not found through electronic research on the databases stated above. Two authors (Z.H. and N.Y.) conducted all literature searches, collected the abstracts, separately reviewed the abstracts, and rated the suitability of the articles for inclusion according to the inclusion criteria as age-matched case control without any systemic or ocular diseases, including a group of diagnosed idiopathic PD.  Figure 1 represents the flowchart of the search strategy. A total of 10 studies were finally included with one study of Nassar et al. [31], which examined two different groups of PDs: one including those newly diagnosed patients with no history of drug usage and the second one including those who received anti-Parkinsonian drugs.

### 2.1. Quality Assessment

Quality scoring of the included studies was carried out using the Newcastle–Ottawa Scale (NOS), applicable to assess the quality of the case-control studies checklists. This scale consists of three different parts, including selection, comparability, and exposure. The selection part consists of four questions: (1) case definition: to evaluate whether there is a distinct definition of the issue, (2) case representation: to evaluate whether there is consecutive or clear case representative series, (3) control selection: to evaluate whether the control group was selected from the community or hospital, and (4) definition of controls: to evaluate whether there are criteria for control selection. The comparability part consists of one question: to evaluate whether there is a comparability of both cases and controls for the confounders. (1) Exposure ascertainment: to assess whether exposure is blinded or not. (2) Similarity: to examine whether the same method is used for both groups. (3) Nonresponse rate: to compare whether both groups have a similar response rate.  Table 1 summarizes the quality assessment data of the included studies.

### 2.2. Data Collection

The collected data included the first author's name, publication year, country, age, sample size, duration of the disease, diagnosed according to established diagnostic criteria (e.g., United Kingdom PD Society Brain Bank [UK-PDSBB]), and stage of PD. Outcome variables included were as follows: mean foveal, parafoveal, and perifoveal retinal thickness (temporal, superior, nasal, and inferior); RNFL thickness (average, temporal, superior, nasal, and inferior); ganglion cell complex (GCC) thickness (average, superior, and inferior); latency (P100 and N75); and amplitude of VEP.  Table 2 summarized the excluded data from articles.

### 2.3. Statistical Considerations

For each study, the difference in mean of the changes in the time latency, amplitude, and choroidal/retinal thickness between treatment and control groups was used as the main effect size. To pool the effect sizes across studies, a random-effects model was used. Heterogeneity was evaluated by the Cochrane *Q* test (the significance level was considered to be 0.05) and the *I*^2^ index. Publication bias was evaluated graphically by funnel plots and statistically by the Egger's regression intercept method. Subgroup analysis was performed to explore the effect of duration and correlation between RNFL and P100 latency; all statistical analyses were performed using Comprehensive Meta-Analysis (Version 2, Biostat Inc., USA).

## 3. Results

Ten studies met our inclusion criteria, of which 337 PD patients and 273 healthy control subjects were enrolled in this meta-analysis. The age range of the participants was 30–80 years old.

### 3.1. Pattern VEP

**Figure 2:**
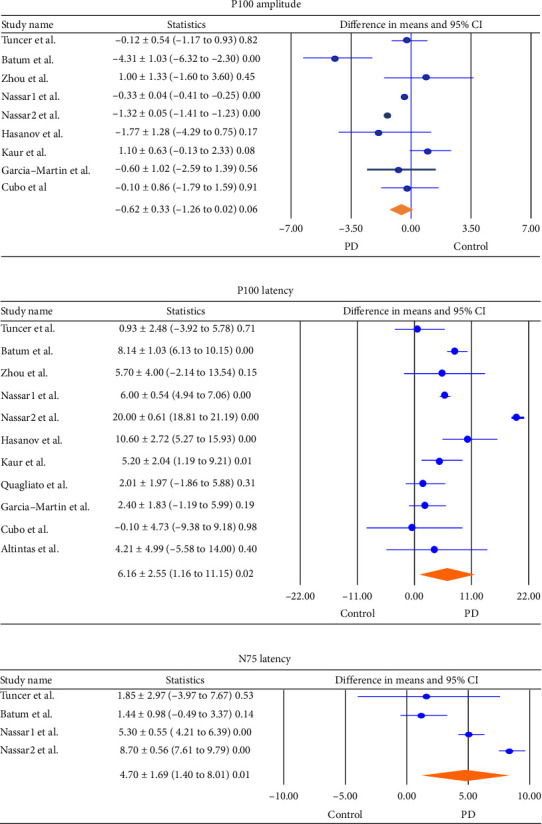
Meta-analysis of the difference in means (a). P100 amplitude (b). P100 latency (c). N75 latency in the eyes of the patients with Parkinson's disease and the healthy group (difference in means ± SD [lower/upper limit], *p* value). Horizontal bars indicate the 95% confidence interval. Numbers in the total indicate the assessed eyes, for which a difference in means was estimable. CI: confidence interval; PD: Parkinson's disease.

There was no statistically significant difference in the pooled difference in means of P100 amplitude between the two groups. The pooled difference in means of P100 amplitude is −0.62 ± 0.33 microvolts (μv) ([−1.26 to –0.02], *p*=0.06) in the PD group compared to the control group (Figure 2(a)) (Egger's intercept was −4.13) (*p*=0.47 ± 4.72). In contrast, the pooled difference in means of the P100 latency between the two groups indicated a significant difference of 6.16 ± 2.55 s (sec) ([1.16 to 11.15], *p*=0.02) (Egger's intercept was −3.36) (*p*=0.28 ± 2.94), with higher latency times observed in PD patients compared to controls (Figure 2(b)). In addition, the pooled difference in means of N75 latency time was significantly higher in PD patients compared to controls, with a difference of 4.70 ± 1.69 s ([1.40 to 8.01], *p*=0.01) (Egger's intercept was −4.13) (*p*=0.99± 2.56) (Figure 2(c)).

### 3.2. OCT

**Figure 3:**
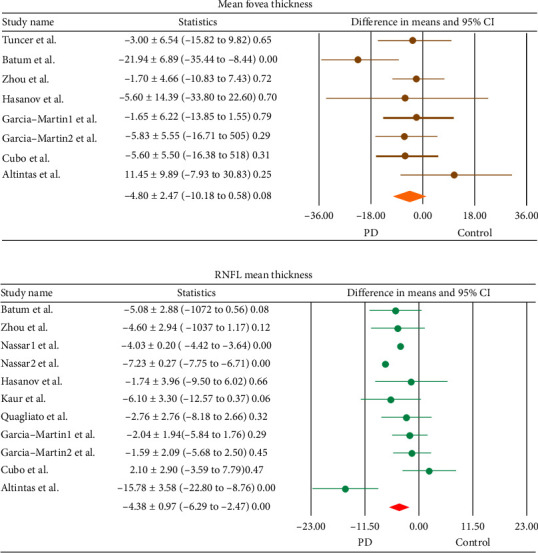
Meta-analysis of the difference in (a). Mean fovea thickness (b). RNFL thickness of the eyes of the patients with Parkinson's disease and the healthy group (difference in means ± SD [lower/upper limit], *p* value). Horizontal bars indicate the 95% confidence interval. RNFL: retinal nerve fiber layer.

Pooled difference in means of foveal thickness was 4.80 ± 2.74 μm (μm) ([−10.18 to 0.58] *p*=0.08) (Egger's intercept was 0.27 (*p*=0.87 ± 1.62), which was lower in the PD subjects compared to healthy subjects (Figure 3(a)); however, the difference did not reach the statistically significance level.  Figure 3(b) showed that there was a statistically significant pooled difference in means of the RNFL thicknesses between the two groups with a thinner RNFL layer, 4.38 ± 0.97 μm, in PD compared to healthy subjects ([−6.29 to −2.47], *p* < 0.001) (Egger's intercept was 0.27) (*p*=0.83 ± 1.29).

Further analysis considering changes in different quadrants revealed that the pooled difference in means of parafovea and perifovea thickness was significantly different in the superior of the parafovea and nasal of the perifovea with 8.15 ± 3.20 and 5.33 ± 1.96 μm thinness (*p*=0.01) in PD subjects compared to healthy subjects (Table 3). According to Table 4, there was a significant difference in means of the peripapillary RNFL thickness in four quadrants of temporal (−4.79 ± 1.40), superior (−5.69 ± 1.45), nasal (−3.79 ± 0.61), and inferior (−5.16 ± 2.30) in PD patients compared to the control (*p* < 0.05), with thinner peripapillary RNFL layer in PD patients.

### 3.3. Subgroup Analysis

#### 3.3.1. VEP

**Figure 4:**
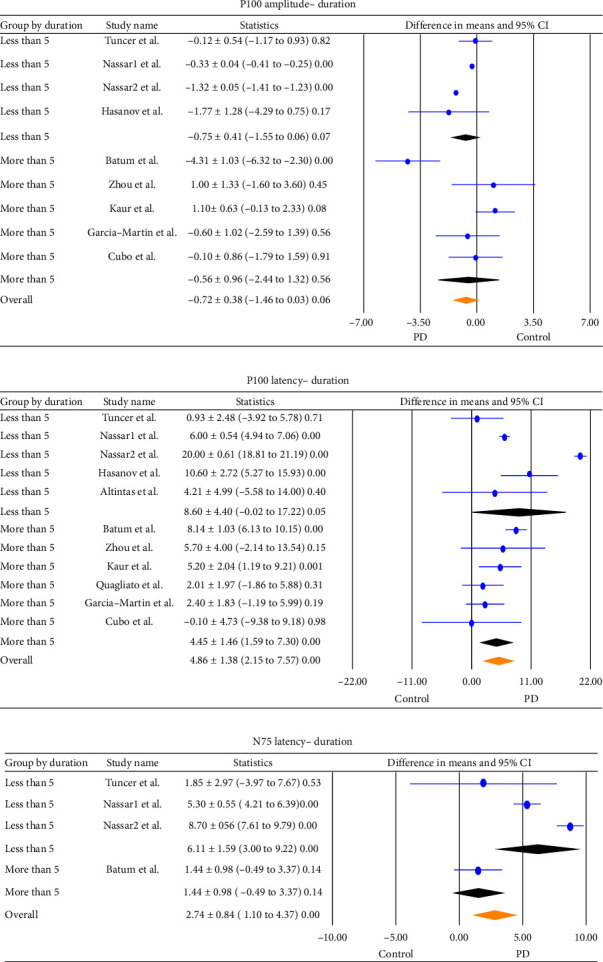
Subgroup analysis for the PD duration of the pooled difference in means in (a). P100 amplitude (b). P100 latency (c). N75 latency (difference in mean ± SD [lower/upper limit],  *p* value). Horizontal bars indicate the 95% confidence interval. Numbers in the total indicate the assessed eyes, for which a difference in means was estimable. CI: confidence interval; PD: Parkinson's disease.

A subgroup analysis accounting for the PD duration showed that although the P100 amplitude was lower in both groups of less/more than 5 years of disease, the pooled difference was not statistically significant (−0.75 ± 0.41 [−1.75, 0.06], *p*=0.07 and −0.56 ± 0.96 [−2.44–1.32], *p*=0.56, respectively) (Figure 4(a)). Moreover, subgroup analysis for the disease duration revealed a statistically significant pooled difference in means of P100 latency between the two participants in either group of less than 5 years (8.60 ± 4.40 [−0.02 to 17.22], *p*=0.05) and more than 5 years of disease duration (4.86 ± 1.38 [−1.59–7.30], *p* ≤ 0.001) (Figure 4(b)). The results of subgroup analysis for the duration of N75 latency revealed a significant pooled difference in means (6.11 ± 1.59 [3.00 to 9.22]. *p* < 0.001) between PD patients and the control group in the group with less than 5 years of disease duration, but no significant difference with more than 5 years of disease duration (1.44 ± 0.98 [−0.49 to 3.37], *p*=0.14) (Figure 4(c)).

#### 3.3.2. OCT

**Figure 5:**
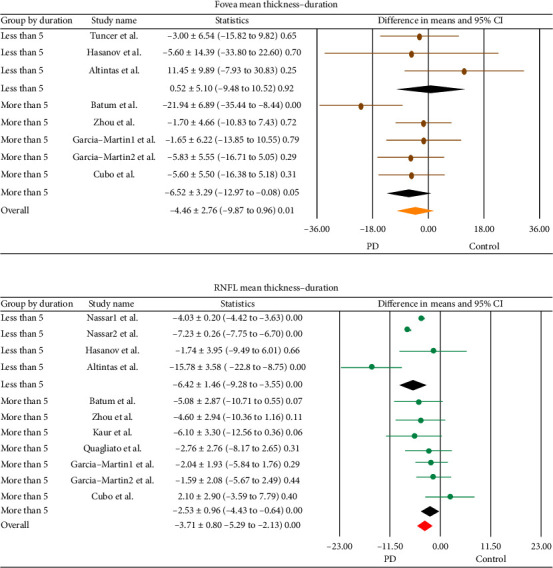
Subgroup analysis for the PD duration of the pooled difference in means in (a). Mean fovea thickness (b). RNFL mean thickness (difference in mean ± SD [lower/upper limit], *p* value). Horizontal bars indicate the 95% confidence interval. Numbers in the total indicate the assessed eyes, for which a difference in means was estimable. CI: confidence interval; PD: Parkinson's disease.

The results of subgroup analysis accounting for disease duration showed that there were no statistically significant differences in foveal thickness between PD patients and healthy controls in either less (−0.52 ± 5.10, *p*=0.92) or more (−6.52 ± 3.29, *p*=0.05) than 5 years of disease duration (Figure 5(a)). However, subgroup analysis considering disease duration revealed that there were significant differences in mean RNFL thickness between PD patients and healthy controls in both groups of less (−6.42 ± 1.46, *p* < 0.001) and more (−2.53 ± 0.96, *p* < 0.001) than 5 years of disease duration (Figure 5(b)).

### 3.4. Correlation

**Figure 6:**
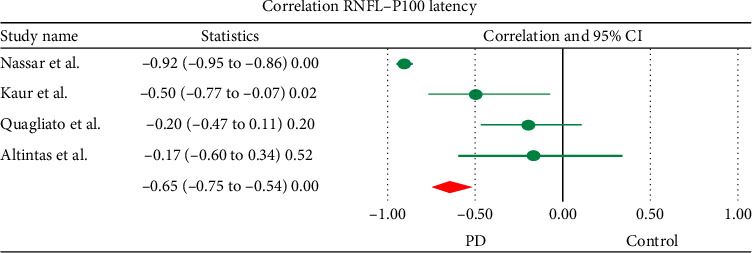
Correlations between RNFL thickness and P100 latency data in PD patients (correlation [lower/upper limit],  *p* value). Horizontal bars indicate the 95% confidence interval. Numbers in the total indicate the assessed eyes, for which a difference in means was estimable. CI: confidence interval; PD: Parkinson's disease.

Correlation analysis revealed that there was a statistically significant negative correlation between RNFL weighted mean difference (PD versus control) and P100 latency (*r* = −0.65, *p*≤ 0.001).  Figure 6 represents the related results of meta-correlation analysis.

## 4. Discussion

This systematic review and meta-analysis aimed to investigate alterations in VEPs and OCT findings in patients with PD compared to healthy controls. While previous meta-analyses [14, 32–35] investigated the changes in retinal layer thickness and VEP responses, the current meta-analysis included studies which investigated changes in VEP responses and OCT findings of the unique participants, who underwent both VEP and OCT tests. Our findings showed a general decrease in posterior layer thickness, which was accompanied by an increase in the latency and a decrease in the amplitude of the VEP response.

Our analysis indicated that the P100 amplitude of VEP was lower in PD patients compared to the control group, although this difference did not reach statistical significance. Furthermore, both P100 and N75 latencies were longer in PD patients than in controls, consistent with findings from previous studies [26, 31, 36–38]. The prolonged VEP latencies observed in PD patients are believed to be linked to neurotransmitter deficiencies rather than demyelinating diseases [39, 40]. Notably, VEP latency is considered less influenced by dopaminergic medications and a more sensitive indicator of foveal electrical activity [38]. While P100 latency is commonly used to detect visual pathway abnormalities in PD patients, some studies [25, 29, 41–43] did not find significant changes in P100 latency, possibly due to variations in VEP stimulation, sample sizes, or disease severity levels. A 6-ms increase in P100 wave latency may suggest visual pathway pathology based on normal values [44]. Subgroup analyses revealed a significant increase in P100 latency with longer durations of PD, indicating early functional effects of the disease [29, 31, 38]. In addition, our findings demonstrated significantly higher N75 latencies in PD groups compared to controls, particularly in relation to disease duration. Contrary to latency findings, there was no noticeable difference in P100 amplitude between PD patient groups based on disease duration, consistent with previous research. These results align with existing literature that has not found a correlation between P100 amplitude and disease duration [14, 25, 26, 29, 42].

The function of the retinal inner plexiform layer and horizontal cells may be affected by degeneration of dopaminergic neurons and decreased dopamine in PD patients, which potentially disrupts the transmission of visual signals and accounts for VEP abnormalities [45]. This inference is partially supported by the thinning of intraretinal layers observed on OCT in patients with PD [29]. Our investigations revealed no significant difference in fovea and parafovea and perifovea thickness between the two groups, and most studies robustly support this result [25, 26, 37, 42, 43]. Foveal thickness has been found to be thinner in the Parkinson's group, which is in contradiction to the Atintas et al.'s [43] findings. Previous studies [29, 30] have shown that the decrease in macular area thickness in PD occurs primarily in the inner layers. Subgroup analysis showed that there is an increase in retinal changes with increasing disease duration, which is parallel with the findings of Nassar et al.'s study [31].

There was a considerable thinning in retinal layers especially the RNFL layer in PD patients compared to the healthy subjects, which could be due to only unmyelinated axons in the RNFL [46–48]. Our findings are in parallel with studies which stated that measuring the RNFL thickness may also be a means of tracking axonal loss in PD patients [29, 31, 38, 43]. Assuming that the normal age-related decay of RNFL thickness is 92.2 μm [49, 50], some studies reported that the RNF layer was clinically thinner in the PD patients [30, 31, 38]; however, other studies revealed no significant difference in RNFL thickness between PD patients and healthy subjects [25, 26, 29, 37, 41–43, 51].

Moreover, thinning of the RNFL has been documented in various neurodegenerative disorders such as multiple sclerosis and Alzheimer's disease [52], which implies that retinal degeneration may transpire concurrently with central neurodegenerative alterations [53]. Some studies yielded contradictory results [25, 26, 30, 41, 42] and found no reduction in RNFL thickness, which can be due to the short duration of the disease. RNFL thinning in the four quadrants, which mainly occurred in the temporal regions, is consistent with the pattern of P-cells distribution [54]. This pattern is similar to what is described for mitochondrial optic neuropathies, where typically the pathology affects the papillomacular bundle and is hallmarked by the preferential loss of the P-cells, leading to temporal pallor of the optic disc and a central visual field [55, 56]. Subgroup analysis revealed a significant RNFL thinning with respect to the history of PD, either less than or more than 5 years. Our results are consistent with Nassar et al.'s [31] study; however, in studies [38, 41, 43], no significant decrease in the thickness of the RNFL was observed with the increase in the history of the disease, possibly because their patients were in mild-to moderate stages of disease [38, 41]. Similarly, in Altintas et al.'s study, the loss of dopaminergic neurons has no discernible effect on the thickness of RNFL [43]. The gradual apoptosis of the retinal ganglion cells may be associated with the slow degeneration of dopaminergic neurons in the amacrine cells and retinal ganglion cells, which was found to be the most prominent RNFL thinning in the superior and inferior quadrants of PD patients [52]. The results correlation analysis showed that the weighted mean difference of mean RNFL was negatively associated with P100 latency, which indicates that the thinning of the RNFL layer leads to an increase in the latency of the P100 wave. Thinning of the RNFL layer in PD patients has been consistently linked with the increasing delay in P100 response [31, 38]; however, some studies reported opposite findings [41, 43]. The axons of ganglion cells are represented by the RNFL, and poor dopaminergic input to the ganglion cells causes atrophy of these particular fibers, which is reflected as thinning of the RNFL [36]. The main strength of the present meta-analysis is including studies which reported the changes of the VEP responses and OCT features in the same patient, which increases the precision and comparability of the results and also analyzes and interprets changes in all existing parameters. However, this meta-analysis was limited by a small number of studies that investigated the changes in either GCL or GCC, nonhomogeneous disease severity scales, and no further detailed information regarding medications. Publication bias is also a coincidence in all meta-analysis studies.

In summary, patients with PD demonstrated a significant delay in VEP response latency compared to the control cohort, with no substantial variance in VEP amplitude. In addition, OCT findings indicated a notable reduction in RNFL thickness among PD patients. Both VEP and OCT assessments hold promise as valuable clinical tools for aiding in disease diagnosis, grading, and importantly, assessing the effectiveness of treatment interventions and monitoring patient progress.

## Figures and Tables

**Table 1:** Quality assessment data of the included studies.

Study	Selection				Comparability	Exposure		
	Case definition	Case representation	Control definition/selection			Exposure ascertainment	Same method	Nonresponse rate
Tuncer et al.	+	+	−	ND	+	−	+	+
Batum et al.	+	+	+	+	+	−	+	+
Zhou et al.	+	+	+	+	+	−	+	+
Nassar et al.	+	+	+	ND	+	−	+	+
Hasanov et al.	+	+	+	ND	+	−	+	+
Kuar et al.	+	+	+	ND	+	−	+	+
Quagliato et al.	+	+	+	−	+	−	+	+
Garcia-Martin et al.	+	+	+	−	+	−	+	+
Cubo et al.	+	+	+	ND	+	−	+	+
Altintas et al.	+	+	+	ND	+	−	+	+

*Note:* Case definition: independent validation. Case representation: consecutive or obviously representative series of cases. Control definition: no history of disease. Control selection: how to choose healthy people. Comparability: study controls for age. Exposure ascertainment: written self-report or medical record only. Same method of ascertainment for cases and controls. Same rate for both groups.

Abbreviation: ND, not described.

**Table 2:** Extracted data of the included studies.

RNFL temporal/RNFL nasal (mean ± SD)	RNFL inferior (mean ± SD)	GCC (mean ± SD)	GCC superior/GCC inferior (mean ± SD)		
PD = 81 (58–111)/70 (55–95) *C* = 90 (63–142)/80 (60–90)	PD = 133 (101–171)/134 (102–157) *C* = 127 (104–257)/135 (111–172)	NA	PD = 96.57 ± 10.16/97.28 ± 10.11 *C* = 98.57 ± 7.45/99.92 ± 7.88		
PD = 59.23 ± 15.13/67.21 ± 16.46 *C* = 62.57 ± 12.26/72.97 ± 13.4	PD = 108.79 ± 22.96/110.19 ± 19.67 *C* = 109.90 ± 18.37/110.90 ± 20.39	PD = 65.85 ± 20.73 *C* = 79.50 ± 4.00	NA		
PD = 70.4 ± 17.1/67.0 ± 10.2 *C* = 71.2 ± 10.2/70.0 ± 8.0	PD = 114.7 ± 15.8/120.9 ±20.1 *C* = 120.0 ± 19.0/130.8 ± 18.2	PD = 80.5± 5.6 *C* = 82.3 ±6.2	PD = 80.3 ± 6.2/78.4 ± 6.3 *C* = 82.3 ± 8.0/81.6 ± 6.3		
PD1 = 68.2 ± 1.22/72.6 ± 1.26 PD2 = 63.08 ± 1.56/72.83 ± 0.83 *C* = 73 ± 1.15/76.6 ± 1.95	PD1 = 111.1 ± 0.73/106.7 ± 0.99 PD2 = 106.9 ± 1.62/98.5 ± 1.62 *C* = 117.4 ± 1.43/110.4 ± 1.07	PD = 69.7 ± 22.3 *C* = 82.2 ± 5.0	PD = 66.0 ± 20.6/71.5 ± 15.8 *C* = 80.6 ± 6.5/81.5 ± 5.3		
PD = 73.89 ± 12.86/83.13 ± 19.00 *C* = 70.32 ± 7.46/83.89 ± 13.94	PD = 112.97 ± 21.84/118.61 ± 21.58 *C* = 123.32 ± 15.05/119.82 ± 13.92	NA	NA		
PD = 51.2 ± 15.1/72.8 ± 18.0 *C* = 58.0 ± 5.6/70.2 ± 8.7	PD = 106.7 ± 20.1/110.2 ± 23.4 *C* = 116.7 ± 13.7/119.4 ± 15.2	NA	NA		
NA	NA	NA	NA		
PD: = 62.19 ± 10.5/74.95 ± 10.3 *C* = 66.61 ± 10.7/75.61 ± 12.6	PD: = 113.33 ± 13.0/119.57 ± 23.7 *C* = 115.80 ± 12.6/121.41 ± 18.0	NA	NA		
PD = 68.16 11.2/78.74 ± 14.6 *C* = 72.05 ± 9.6 /79.35 ± 15.5	PD = 116.53 ± 12.7/127.37 ± 18.7 *C* = 118.73 ± 14.7/128.33 ± 20.0	NA	NA		
NA	NA	NA	NA		
PD = 70.88 ± 18.12/75.94 ± 19.07 *C* = 71.90 ± 16.73/99.45 ± 36.85	PD = 125.47 ± 24.41/126.26 ± 35.36 *C* = 138.77 ± 17.73/142.72 ± 19.78	NA	NA		

**Parafovea superior/parafovea inferior (mean ± SD)**	**Perifovea temporal/perifovea nasal (mean ± SD)**	**Perifovea superior/perifovea inferior (mean ± SD)**	**RNFL mean thickness (mean ± SD)**		

PD = 317 (280–355)/314 (237–380) *C* = 319 (290–362)/314 (259–363)	PD = 289 (244–326)/303 (266–345) *C* = 283 (235–320)/310 (274–344)	PD = 288 (262–320)/PD = 285 (249–342) *C* = 288 (258–312)/291 (233–309)	NA		
PD = 306.52 ± 35.43/308.21 ± 25.26 *C* = 323.43 ± 21.52/316.30 ± 20.49	PD = 253.42 ± 37.65/282.94 ± 22.21 *C* = 262.40 ± 12.19/293.80 ± 19.16	PD = 266.77 ± 19.7/258.00 ± 28.29 *C* = 279.83 ± 12.13/264.43 ± 14.54	PD = 85.79 ± 18.29 *C* = 90.87 ± 8.88		
PD = 314.9 ± 16.8/313.7 ± 16.2 *C* = 316.5 ± 11.5 /314.8 ± 13.8	PD = 257.5 ± 13.7/290.8 ± 15.1 *C* = 258.0 ± 10.3/294.0 ± 12.9	PD = 271.9 ± 12.0/263.2 ± 14.4 *C* = 274.7 ± 12.5/262.9 ± 11.0	PD = 93.4 ± 10.7 *C* = 98.0 ± 9.4		
NA	NA	NA	PD1 = 90.30 ± 0.73 PD2 = 87.1 ± 1.09 *C* = 94.33 ± 0.55		
NA	NA	NA	PD = 96.89 ± 15.13 *C* = 98.63 ± 8.29		
NA	NA	NA	PD = 85.0 ± 12.4 *C* = 91.1 ± 8.0		
NA	NA	NA	PD: = 103.09 ± 12.04 *C* = 105.85 ± 12.82		
PD: = 319.48 ± 14.3/314.98± 14.4 *C* = 322.86 ± 17.5/318.38 ± 17.6	PD: = 265.00 ± 16.6/289.25 ± 21.8 *C* = 266.00 ± 20.2/291.33 ± 17.7	PD: = 277.13 ± 16.4/267.00 ± 16.9 *C* = 277.95 ± 17.3/263.05 ± 17.9	PD: = 92.62 ± 8.5 *C* = 94.66 ± 8.5		
PD: = 331.95 ± 16.4/327.68 ± 16.6 *C* = 335.89 ± 15.6/329.80 ± 18.2	PD: = 277.00 ± 15.9/302.41 ± 17.7 *C* = 278.00 ± 13.7/307.20 ± 20.4	PD: = 287.55 ± 16.8/278.30 ± 21.1 *C* = 289.63 ± 20.7/279.08 ± 16.4	PD = 97.84 ± 8.4 *C* = 99.43 ± 10.1		
PD = 274.2 ± 14.2/275.5 ± 11.8 *C* = 268.9 ± 13.1/271.1 ± 12.9	PD = 220.5 ± 13.6/253.0 ± 12.8 *C* = 214.6 ± 12.8/251.8 ± 16.9	PD = 242.9 ± 15.8/225.4 ± 13.3 *C* = 235.4 ± 15.7/225.0 ± 19.6	PD = 97.7 ± 9.3 *C* = 95.6 ± 12.9		
PD = 268.05 ± 15.76/273.61 ± 19.45 *C* = 277.36 ± 11.18/280.04 ± 12.06	PD = 220.58 ± 10.32/250.38 ± 16.71 *C* = 234.31 ± 14.92/265.31 ± 12.00	PD = 242.35 ± 16.13/227.52 ± 17.06 *C* = 248.81 ± 9.42/240.45 ± 9.97	PD = 98.76 ± 10.90 *C* = 114.54 ± 5.72		

**OCT model**	**P100 amplitude/P100 latency (mean ± SD)**	**N75 latency (mean ± SD)**	**Average foveal thickness (mean ± SD)**	**Parafovea temporal/parafovea nasal (mean ± SD)**	

Optovue	PD = 5.35 ± 2.09/109.23 ± 6.92 *C* = 5.47 ± 1.82/108.3 ± 12.63	PD = 82.97 ± 11.48 *C* = 81.12 ± 13.23	PD = 262 (219–319)^∗^ *C* = 265 (229–327)	PD = 265 (229–327)/320 (261–358) *C* = 312 (257–355)/319 (279–368)	
Carl Zeiss	PD = 10.39 ± 6.08/125.87 ± 4.93 *C* = 14.70 ± 3.98/117.73 ± 5.33	PD = 77.94 ± 5.55 *C* = 76.50 ± 4.18.76	PD: 232.63 ± 42.62 *C*: 254.57 ± 23.61	PD = 292.87 ± 34.55/307.42 ± 33.61 *C* = 306.60 ± 19.91/321.77 ± 21.4	
Zeiss Cirrus	PD = 6.6 ± 5.1/113.3 ± 14.7 *C* = 5.6 ± 3.9/107.6 ± 12.6	NA	PD = 246.0 ± 18.3 *C* = 247.7 ± 13.1	PD = 305.6 ± 16.7/318.8 ± 16.8 *C* = 305.0 ± 11.5/320.3 ± 13.2	
Zeiss Cirrus	PD1 = 5.27 ± 0.09/102.3 ± 1.63 PD2 = 4.28 ± 0.15/116.3 ± 2.13 *C* = 5.6 ± 0.16/96.3 ± 1.88	PD1 = 70.5 ± 1.08 PD2 = 73.9 ± 1.37 *C* = 65.2 ± 2.34	NA	NA	
Topcon	PD:D = 7.03 ± 3.7/113.2 ± 11.5 *C* = 8.8 ± 4.2/102.6 ± 2.9	NA	PD: = 219.6 ± 55.5 *C* = 225.2 ± 29.2	NA	
Carl Zeiss	PD = 9.4 ± 2.3/110.2 ± 8.6 *C* = 8.3 ± 1.6/105 ± 3.1	NA	NA	NA	
Optovue	PD = NA/108.36 ± 10.23 *C* = NA/106.35 ± 6.99	NA	NA	NA	
Zeiss Cirrus	PD = 11.9 ± 4.5/115.3 ± 8.2 *C* = 12.5 ± 4.4/112.9 ± 7.8	NA	PD: = 257.45 ± 25.9 *C* = 259.10 ± 29.1	PD: = 307.38 ± 17.4/320.83 ± 16.1 *C* = 315.95 ± 20.9/326.62 ± 18.4	
Heidelberg			PD: = 270.80 ± 24.2 *C* = 276.63 ± 24.5	PD: = 320.95 ± 15.3/335.42 ± 17.4 *C* = 325.35 ± 17.8/339.35 ± 15.8	
Stratus Carl Zeiss	PD = 6.4 ± 3.3/108.6 ± 23.3 *C* = 6.5 ± 2.9/108.7 ± 4.2	NA	PD = 216.0 ± 21.7 *C* = 221.6 ± 20.9	PD = 265.0 ± 12.8/279.8 ± 13.1 *C* = 261.0 ± 12.3/274.4 ± 14.0	
*C*arl Zeiss	PD = NA/ 113.38 ± 13.75 *C* = NA/109.17 ± 11.42	NA	PD = 208.26 ± 29.78 *C* = 196.81 ± 16.72	PD = 257.00 ± 16.91/270.29 ± 16.52 *C* = 265.86 ± 20.81/275.36 ± 14.55	

**Country**	**Sample size PD/*C***	**Age (mean ± SD)**	**Duration (Y) (mean ± SD)**	**Medication**	**Cognitive function (mean ± SD)**

Turkey	41/29	PD = 65.58 ± 9.89 *C* = 63.48 ± 11.23	2 (0–18)	NA	UPDRS 3 = 19 (6–63) UPDRS Total = 39 (14–109)
Turkey	50/50	PD = 65.10 9.81 *C* = 61.80 ± 5.74	6.45 ± 4.67	Levodopa	UPDRS Total = 45 ± 32
China	24/23	PD = 65.88 ± 6.50 *C* = 63.43 ± 7.11	5.3 ± 4.2	NA	UPDRS 3 = 26.5 ± 12.3 H & Y = 2
Egypt	22/25/20	PD1 = 54.8 6.52 PD2 = 59 ± 3.76 *C* = 57.2 ± 3.76	PD1 = 0.59 ± 0.19 PD2 = 3.59 ± 1.02	PD1: no medication PD2: anti-Parkinsonian drugs	H & Y = PD1 = Stages 1 and 2 PD2 = Stages 2, 3, and 4
Turkey	19/19	PD = 54.39 5.71 *C* = 55.53 ± 6.48	3.93 ± 3.42	Levodopa MAO	H & Y = Stages 1 and 2
India	20/20	PD = 58.6 ± 9.5 *C* = 58.4 ± 9.3	5.8 ± 2.78	NA	H & Y Stages = 2 UPDRS 3 = 19 ± 10.42 (7–51)
Brazil	43/38	PD = 63.1 ± 7.55 *C* = 62.4 ± 7.2	6.98 ± 4.13	Dopaminergic	H & Y 1 and 2 and 3 = mean = 1.78 UPDRS Total = 17.63 ± 8.99
Spain	46/33	PD = 70.72 (55–88) *C* = 69.67 (58–87)	7.7 ± 2.1	Levodopa MAO	H & Y = 2.2 ± 0.8
Spain	30/30	PD = 65.4 ± 8.0 *C* = 62.9 ± 5.5	8.2 ± 4.7	Levodopa	H & Y = 2 (1–4)
Turkey	17/11	PD = 59.2912.8 *C* = 58.09 ± 7.25	4.58 ± 0.86	Levodopa	UPDRS Total = 24.23 ± 3.53

**Study**	**Year**				

Tuncer et al.	2023				
Batum et al.	2022				
Zhou et al.	2021				
Nassar et al.	2020				
Hasanov et al.	2019				
Kaur et al.	2015				
Quagliato et al.	2014				
Garcia-Martin et al.	2014				
Cubo et al.	2014				
Altintas et al.	2008				

Abbreviations: C, control; H & Y, Hoehn & Yahr; MAO, monoamine oxidase inhibitors; NA, not available; OCT, optical coherence tomography; PD, Parkinson's disease; RNFL, retinal nerve fiber layer; SD, standard deviation; VEP, visual evoked potential.

^∗^Median; all OCT data in micrometers and VEP data in microseconds.

**Table 3:** Meta-analysis of the difference in means of the fovea thickness in the eyes of the Parkinson patients compared to the eyes of healthy controls.

	Difference in means	Standard error	Lower limit	Upper limit	*p* value
Parafovea temporal	−11.12	7.07	−24.96	2.73	0.12
Parafovea superior	−8.15	3.20	−14.44	−1.86	**0.01**
Parafovea nasal	−2.60	2.22	−6.96	1.76	0.24
Parafovea inferior	−1.00	1.50	−3.95	1.95	0.50
Perifovea temporal	−1.21	2.50	−6.12	3.70	0.63
Perifovea superior	−2.65	2.60	−7.74	2.44	0.31
Perifovea nasal	−5.33	1.96	−9.17	−1.48	**0.01**
Perifovea inferior	−2.09	1.99	−5.99	1.81	0.29

*Note:* Bold values represents the significant difference.

**Table 4:** Meta-analysis of the difference in means of the peripapillary retinal nerve fiber layer thickness in four quadrants in patients compared to the control.

	Difference in means	Standard error	Lower limit	Upper limit	*p* value
RNFL temporal	−4.79	1.40	−7.54	−2.03	**0.001**
RNFL superior	−5.69	1.45	−8.52	−2.85	**p** ≤ 0.001
RNFL nasal	−3.79	0.61	−4.99	−2.60	**p** ≤ 0.001
RNFL inferior	−5.16	2.30	−9.66	−0.66	**0.02**

*Note:* Bold values represent the significant difference.

Abbreviation: RNFL, retinal nerve fiber layer.

## Data Availability

The data are available from corresponding author (N.Y.) on reasonable request.

## References

[1] Jankovic J. (2008). Parkinson’s Disease: Clinical Features and Diagnosis. *Journal of Neurology, Neurosurgery and Psychiatry*.

[2] Hamani C., Lozano A. M. (2003). Physiology and Pathophysiology of Parkinson’s Disease. *Annals of the New York Academy of Sciences*.

[3] Pagonabarraga J., Kulisevsky J., Strafella A. P., Krack P. (2015). Apathy in Parkinson’s Disease: Clinical Features, Neural Substrates, Diagnosis, and Treatment. *The Lancet Neurology*.

[4] Dorsey E., Sherer T., Okun M. S., Bloem B. R. (2018). The Emerging Evidence of the Parkinson Pandemic. *Journal of Parkinson’s Disease*.

[5] Chen Y., Zhu G., Liu D. (2020). The Morphology of Thalamic Subnuclei in Parkinson’s Disease and the Effects of Machine Learning on Disease Diagnosis and Clinical Evaluation. *Journal of the Neurological Sciences*.

[6] Guo L., Normando E. M., Shah P. A., De Groef L., Cordeiro M. F. (2018). Oculo‐Visual Abnormalities in Parkinson’s Disease: Possible Value as Biomarkers. *Movement Disorders*.

[7] Postuma R. B., Aarsland D., Barone P. (2012). Identifying Prodromal Parkinson’s Disease: Pre‐Motor Disorders in Parkinson’s Disease. *Movement Disorders*.

[8] Zhang Y., Zhang X., Yue Y., Tian T. (2022). Retinal Degeneration: A Window to Understand the Origin and Progression of Parkinson’s Disease?. *Frontiers in Neuroscience*.

[9] Mailankody P., Lenka A., Pal P. K. (2019). The Role of Optical Coherence Tomography in Parkinsonism: A Critical Review. *Journal of the Neurological Sciences*.

[10] Borm C. D., Visser F., Werkmann M. (2020). Seeing Ophthalmologic Problems in Parkinson Disease: Results of a Visual Impairment Questionnaire. *Neurology*.

[11] Spaide R. F., Fujimoto J. G., Waheed N. K., Sadda S. R., Staurenghi G. (2018). Optical Coherence Tomography Angiography. *Progress in Retinal and Eye Research*.

[12] Armstrong R. A. (2008). Visual Signs and Symptoms of Parkinson’s Disease. *Clinical and Experimental Optometry*.

[13] Lascano A. M., Lalive P. H., Hardmeier M., Fuhr P., Seeck M. (2017). Clinical Evoked Potentials in Neurology: A Review of Techniques and Indications. *Journal of Neurology, Neurosurgery and Psychiatry*.

[14] He S. B., Liu C. Y., Chen L. D. (2018). Meta-Analysis of Visual Evoked Potential and Parkinson’s Disease. *Parkinson’s Disease*.

[15] Liu C., Zhang Y., Tang W., Wang B., Wang B., He S. (2017). Evoked Potential Changes in Patients With Parkinson’s Disease. *Brain and Behavior*.

[16] Petrova M., Angov G., Traykov L. Visual and Brainstem Auditory-Evoked Potentials Correlate With Specific Motor and Non-Motor Symptoms in Parkinson’s Disease.

[17] Murphy N., Killen A., Gupta R. K. (2021). Exploring Bottom-Up Visual Processing and Visual Hallucinations in Parkinson’s Disease With Dementia. *Frontiers in Neurology*.

[18] Delalande I., Hache J., Forzy G., Bughin M., Benhadjali J., Destee A. (1998). Do Visual‐Evoked Potentials and Spatiotemporal Contrast Sensitivity Help to Distinguish Idiopathic Parkinson’s Disease and Multiple System Atrophy?. *Movement Disorders*.

[19] Alves J. N., Westner B. U., Højlund A., Weil R. S., Dalal S. S. (2023). Structural and Functional Changes in the Retina in Parkinson’s Disease. *Journal of Neurology Neurosurgery and Psychiatry*.

[20] Kirbas S., Turkyilmaz K., Tufekci A., Durmus M. (2013). Retinal Nerve Fiber Layer Thickness in Parkinson Disease. *Journal of Neuro-Ophthalmology*.

[21] Ucak T., Alagoz A., Cakir B., Celik E., Bozkurt E., Alagoz G. (2016). Analysis of the Retinal Nerve Fiber and Ganglion Cell: Inner Plexiform Layer by Optical Coherence Tomography in Parkinson’s Patients. *Parkinsonism and Related Disorders*.

[22] Satue M., Garcia-Martin E., Fuertes I. (2013). Use of Fourier-Domain OCT to Detect Retinal Nerve Fiber Layer Degeneration in Parkinson’s Disease Patients. *Eye*.

[23] Bayhan H. A., Aslan Bayhan S., Tanık N., Gürdal C. (2014). The Association of Spectral-Domain Optical Coherence Tomography Determined Ganglion Cell Complex Parameters and Disease Severity in Parkinson’s Disease. *Current Eye Research*.

[24] Garcia-Martin E., Satue M., Fuertes I. (2012). Ability and Reproducibility of Fourier-Domain Optical Coherence Tomography to Detect Retinal Nerve Fiber Layer Atrophy in Parkinson’s Disease. *Ophthalmology*.

[25] Tuncer Z., Dereli Can G., Dönmez Keklikoğlu H., Eren F. A., Yülek F., Deniz O. (2023). The Relationship Between Visual-Evoked Potential and Optic Coherence Tomography and Clinical Findings in Parkinson Patients. *Parkinson’s Disease*.

[26] Zhou M., Wu L., Hu Q., Wang C., Ye J., Chen T. (2021). Visual Impairments Are Associated With Retinal Microvascular Density in Patients With Parkinson’s Disease. *Frontiers in Neuroscience*.

[27] Robbins C., Thompson A., Bhullar P. J. J. O. (2021). Characterization of Retinal Microvascular and Choroidal Structural Changes in Parkinson Disease. *JAMA Ophthalmology*.

[28] Shi C., Chen Y., Kwapong W. R. (2020). Characterization by Fractal Dimension Analysis of the Retinal Capillary Network in Parkinson Disease. *Retina*.

[29] Garcia-Martin E., Rodriguez-Mena D., Satue M. (2014). Electrophysiology and Optical Coherence Tomography to Evaluate Parkinson Disease Severity. *Investigative Opthalmology and Visual Science*.

[30] Batum M., Ak A. K., Arı M. S., Mayali H., Kurt E., Selçuki D. (2022). Evaluation of the Visual System With Visual Evoked Potential and Optical Coherence Tomography in Patients With Idiopathic Parkinson’s Disease and With Multiple System Atrophy. *Documenta Ophthalmologica*.

[31] H Nassar M., A Ragab O., M Al-Malt A., E Nawar A., H Rashed K., Tageldin E. (2020). Evaluation of the Visual Pathway in Parkinson’s Disease Patients. *The Journal of Neurology and Experimental Neuroscience*.

[32] Deng Y., Jie C., Wang J., Liu Z., Li Y., Hou X. (2022). Evaluation of Retina and Microvascular Changes in the Patient With Parkinson’s Disease: A Systematic Review and Meta-Analysis. *Frontiers of Medicine*.

[33] Huang L., Zhang D., Ji J., Wang Y., Zhang R. (2021). Central Retina Changes in Parkinson’s Disease: A Systematic Review and Meta-Analysis. *Journal of Neurology*.

[34] Zhou W. C., Tao J. X., Li J. (2021). Optical Coherence Tomography Measurements as Potential Imaging Biomarkers for Parkinson’s Disease: A Systematic Review and Meta‐Analysis. *European Journal of Neurology*.

[35] Chrysou A., Jansonius N. M., van Laar T. (2019). Retinal Layers in Parkinson’s Disease: A Meta-Analysis of Spectral-Domain Optical Coherence Tomography Studies. *Parkinsonism and Related Disorders*.

[36] Zhao Y., Dai W., Liu D. (2022). Quantitative Analysis of Related Parameters of Retinal Nerve Fiber Layer and Ganglion Cell Complex Thickness in Patients With Different Degrees of Parkinson’s Disease. *Aging Clinical and Experimental Research*.

[37] Hasanov S., Demirkilinc Biler E., Acarer A., Akkın C., Colakoglu Z., Uretmen O. (2019). Functional and Morphological Assessment of Ocular Structures and Follow-Up of Patients With Early-Stage Parkinson’s Disease. *International Ophthalmology*.

[38] Kaur M., Saxena R., Singh D., Behari M., Sharma P., Menon V. (2015). Correlation Between Structural and Functional Retinal Changes in Parkinson Disease. *Journal of Neuro-Ophthalmology*.

[39] Bodis‐Wollner I., Yahr M. D., Mylin L., Thornton J. (1982). Dopaminergic Deficiency and Delayed Visual Evoked Potentials in Humans. *Annals of Neurology*.

[40] Bodis-Wollner I., Yahr M. D. (1978). Measurements of Visual Evoked Potentials in Parkinson’s Disease. *Brain*.

[41] Quagliato L. B., Domingues C., Quagliato E. M., Abreu E. B., Kara-Junior N. (2014). Applications of Visual Evoked Potentials and Fourier-Domain Optical Coherence Tomography in Parkinson’s Disease: A Controlled Study. *Arquivos Brasileiros de Oftalmologia*.

[42] Cubo E., López Peña M. J., Diez-Feijo Varela E. (2014). Lack of Association of Morphologic and Functional Retinal Changes With Motor and Non-Motor Symptoms Severity in Parkinson’s Disease. *Journal of Neural Transmission*.

[43] Altintaş O., Işeri P., Ozkan B., Cağlar Y. (2008). Correlation Between Retinal Morphological and Functional Findings and Clinical Severity in Parkinson’s Disease. *Documenta Ophthalmologica*.

[44] Mahjoob M., Heravian Shandiz J., Mirzajani A., Ehsaei A., Jafarzadehpur E. (2019). Normative Values of Visual Evoked Potentials in Northeastern of Iran. *Journal of Optometry*.

[45] Büttner T., Kuhn W., Müller T., Heinze T., Pühl C., Przuntek H. (1996). Chromatic and Achromatic Visual Evoked Potentials in Parkinson’s Disease. *Electroencephalography and Clinical Neurophysiology: Evoked Potentials Section*.

[46] Albrecht P., Müller A. K., Südmeyer M. (2012). Optical Coherence Tomography in Parkinsonian Syndromes. *PLoS One*.

[47] Archibald N. K., Clarke M. P., Mosimann U. P., Burn D. J. (2011). Retinal Thickness in Parkinson’s Disease. *Parkinsonism and Related Disorders*.

[48] Bodis-Wollner I. (2009). Retinopathy in Parkinson Disease. *Journal of Neural Transmission*.

[49] Parikh R. S., Parikh S. R., Sekhar G. C., Prabakaran S., Babu J. G., Thomas R. (2007). Normal Age-Related Decay of Retinal Nerve Fiber Layer Thickness. *Ophthalmology*.

[50] Celebi A. R. C., Mirza G. E. (2013). Age-Related Change in Retinal Nerve Fiber Layer Thickness Measured With Spectral Domain Optical Coherence Tomography. *Investigative Opthalmology and Visual Science*.

[51] Miri S., Glazman S., Mylin L., Bodis-Wollner I. (2016). A Combination of Retinal Morphology and Visual Electrophysiology Testing Increases Diagnostic Yield in Parkinson’s Disease. *Parkinsonism and Related Disorders*.

[52] Moreno-Ramos T., Benito-León J., Villarejo A., Bermejo-Pareja F. (2013). Retinal Nerve Fiber Layer Thinning in Dementia Associated With Parkinson’s Disease, Dementia With Lewy Bodies, and Alzheimer’s Disease. *Journal of Alzheimer’s Disease*.

[53] Marchesi N., Fahmideh F., Boschi F., Pascale A., Barbieri A. (2021). Ocular Neurodegenerative Diseases: Interconnection Between Retina and Cortical Areas. *Cells*.

[54] La Morgia C., Ross-Cisneros F. N., Sadun A. A., Carelli V. (2017). Retinal Ganglion Cells and Circadian Rhythms in Alzheimer’s Disease, Parkinson’s Disease, and Beyond. *Frontiers in Neurology*.

[55] La Morgia C., Di Vito L., Carelli V., Carbonelli M. (2017). Patterns of Retinal Ganglion Cell Damage in Neurodegenerative Disorders: Parvocellular vs Magnocellular Degeneration in Optical Coherence Tomography Studies. *Frontiers in Neurology*.

[56] Yu J.-G., Feng Y.-F., Xiang Y. (2014). Retinal Nerve Fiber Layer Thickness Changes in Parkinson Disease: A Meta-Analysis. *PLoS One*.

